# A One Year Follow-Up Study of Natural Killer and Dendritic Cells Activities in Multiple Sclerosis Patients Receiving Glatiramer Acetate (GA)

**DOI:** 10.1371/journal.pone.0062237

**Published:** 2013-04-22

**Authors:** Rune A. Høglund, Trygve Holmøy, Hanne F. Harbo, Azzam A. Maghazachi

**Affiliations:** 1 Department of Physiology, Institute of Medical Basal Sciences, University of Oslo, Oslo, Norway; 2 Department of Neurology, Akershus University Hospital and Institute of Clinical Medicine, University of Oslo, Oslo, Norway; 3 Department of Neurology, Ullevål, Oslo University Hospital and Institute of Clinical Medicine, University of Oslo, Oslo, Norway; Centre de Recherche Public de la Santé (CRP-Santé), Luxembourg

## Abstract

**Background:**

Multiple sclerosis (MS) is a chronic inflammatory, demyelinating and neurodegenerative disease. It is thought to be mediated by CD4^+^ Th1/Th17 cells. More recently, cells of the innate immune system such as dendritic cells (DCs) and natural killer (NK) cells have been in focus. Glatiramer acetate (GA) is an approved drug for treating MS patients.

**Methodology/Principal Findings:**

In the current study we examined the activities of NK and DCs in nine relapsing remitting MS patients for up to one year after initiation of GA treatment. We observed that NK cells isolated from most of these patients have increased cytotoxic activity against K562 cells. Further analysis showed that the same NK cells lysed both autologous immature (i) and mature (m) DCs. In most patients this increased activity was correlated with increased NK cell activating cytotoxicity receptors such as NKp30, NKp44, NKp46 and NKG2D, and reduced expression of the inhibitory molecule CD158 on the surface of these NK cells. The expression of HLA-DR was increased on iDCs and mDCs in the majority of the patients, but no consistency was observed for the expression of HLA-I or HLA-E. Also, the co-stimulatory receptors CD80, CD83 or CD86 expression was down-regulated on iDCs and mDCs in most cases. Further, the expression of CCR6 was increased on mDCs at later time points of therapy (between 32–48 weeks).

**Conclusions/Significance:**

Our results are the first showing the effects of GA treatment on NK cells in MS patients, which may impact future use of this and other drugs to treat this disease.

## Introduction

Cells of the innate immune system include NK cells, that have several important functions such as regulation of the adaptive immune response by secreting cytokines and chemokines [1], and defense against viral infection as well as lysing and killing tumor cells [2]. The innate immune system also comprises dendritic cells (DCs) subsets. Factors such as GM-CSF and type I IFNs or IL-4, released early after interaction between innate immune cells and pathogens, represent potential natural mediators of differentiation and maturation of monocytes into immature DCs (iDCs), and in turn further differentiation into mature cells [3], [4]. It has been observed that myeloid DCs may accumulate in the CNS during experimental autoimmune encephalomyelitis (EAE), where they present myelin autoantigens to CD4^+^ T cells that can differentiate into Th17 cells [5]. Several studies have shown that NK cells and DCs interact in a bidirectional way, which involves cell-to-cell contact. One outcome of this interaction is the ability of activated NK cells to lyse iDCs [6]. How, where and why these two innate immune system cells interact is still unclear, although it has been suggested that such interaction might take place at inflammatory sites [7].

Glatiramer acetate (GA; commercial name Copaxone®) is a synthetic compound made up of the four amino acids (Glu, Ala, Lys, Tyr) that are most common in myelin basic protein [8]. GA is a first-line immunomodulatory therapy in relapsing remitting multiple sclerosis (RRMS) [9]. Although the drug is not as effective as second line therapies like natalizumab and fingolimod, GA is widely used due to few serious side effects. GA also showed promise as maintenance therapy, when used after more intensive immunosuppression [10]. GA reduces relapses by approximately 30%, and animal studies show prevention of EAE in GA treated animals [11]. Among various effects, GA reduces the responsiveness of monocytes to multiple stimuli, including reactivity to ligands for toll-like receptors (TLRs) and inflammatory cytokines such as interferon-gamma (IFN-γ) and GM-CSF [12]. Monocytes isolated from GA-treated MS patients secrete high amounts of the anti-inflammatory cytokine IL-10 and less of the inflammatory cytokine IL-12 [13]. In EAE, GA activates monocytes type 2 which induce naive T cells to become Th2 cells [14]. It was also reported that GA enhances in vitro killing of autologous and allogeneic human immature and mature monocyte-derived DCs by activated human NK cells [15]. GA also reduces the in vitro number of mature DCs expressing CD83 or HLA-DR but does not affect their expression of CD80, CD86, HLA-I, or CCR7 [15]. Administration of GA into mice ameliorated the EAE clinical scores, and this was associated with high killing of iDCs and mDCs by isolated NK cells from these mice [16]. These results indicate that one possible mechanism of action may be exerted by the ability of GA to activate NK cells to lyse DCs. Hence, NK cells exposed to GA kill both iDCs and mDCs, which may impede presentation of antigens to autoreactive T cells. In the current study we examined the effects of GA on NK cells and DCs isolated from MS patients undergoing GA treatment. In particular, we investigated the expression of NK cell activating and inhibitory cytotoxicity receptors as well as cytolytic ability, and the expression of co-stimulatory and MHC molecules on iDCs and mDCs.

## Materials and Methods

### Human Cells

The study was approved by the Regional Committee for Medical Research Ethics for South East Norway. The patients included approved enrolling in the study by written consents. Peripheral blood was collected from nine RRMS patients at the outpatient clinic, Department of Neurology, Oslo University hospital Ullevål, before and during the first year of treatment with GA (Copaxone®). Blood samples (30–60 ml) were collected before treatment start and 1, 4, 8, 16, 32 and 48 weeks after initiation of GA treatment (Table 1). The patients were followed clinically, and two patients reported small relapses that occurred early in their treatment period. None of the patients stopped GA treatment during the observation period, and they were all considered as responders to the treatment (Table 1). All experiments were performed on fresh blood samples immediately after collection and activation. In some cases the collected blood samples did not yield enough isolated cells to perform all experiments.

**Table 1:** Clinical and demographic data of the relapsing remitting multiple sclerosis patients included in the study.

Clinical data								Blood Sampling	Relapses	
ID	Age	Sex	Years since symptom debut	Debut symptom	OCB^1^	EDSS	New MRi lesions during study	Week^2^	Before study N =	During study
1	37	M	5	ON	+	1	Yes	0, 1, 4, 8, 16, 32, 48	2	week 16
2^3^	57	F	4	ON	+	1	No	0, 1, 4, 8, 16, 32, 48	3	−
3	60	M	11	Sensory	ND	ND	No	0, 1, 4, 16, 32	>2	−
4	54	F	20	ON	+	3	ND	0, 1, 4, 8, 16, 32, 48	>3	−
5	40	M	0	Brainstem	+	3	No	0, 1, 4, 8, 16, 32, 48	1	−
6	49	F	9	Pain, fatigue	+	1,5	No	0, 1, 4, 8, 16, 32, 48	2	−
7	41	F	1	Sensory	+	ND	No	0, 1, 4, 48	2	−
8	34	F	8	ON	−	3	No	0, 1, 16	3	week 4
9	30	F	0	Sensory	+	ND	No	0, 1, 4, 8, 16, 32	1	−

1 Two or more oligoclonal bands in CSF at isoelectric focusing that were not present in serum.

2 Number of weeks after treatment starts when blood samples were taken.

3 Patient 2 had previously used interferon-β1a treatment, the others were treatment naive.

Abbreviations: GA = glatiramer acetate, M = Male, F = Female, ON = Optic Neuritis, EDSS = Expanded disability status scale, OCB = Oligoclonal Bands, ND = No data.

**Figure 1:**
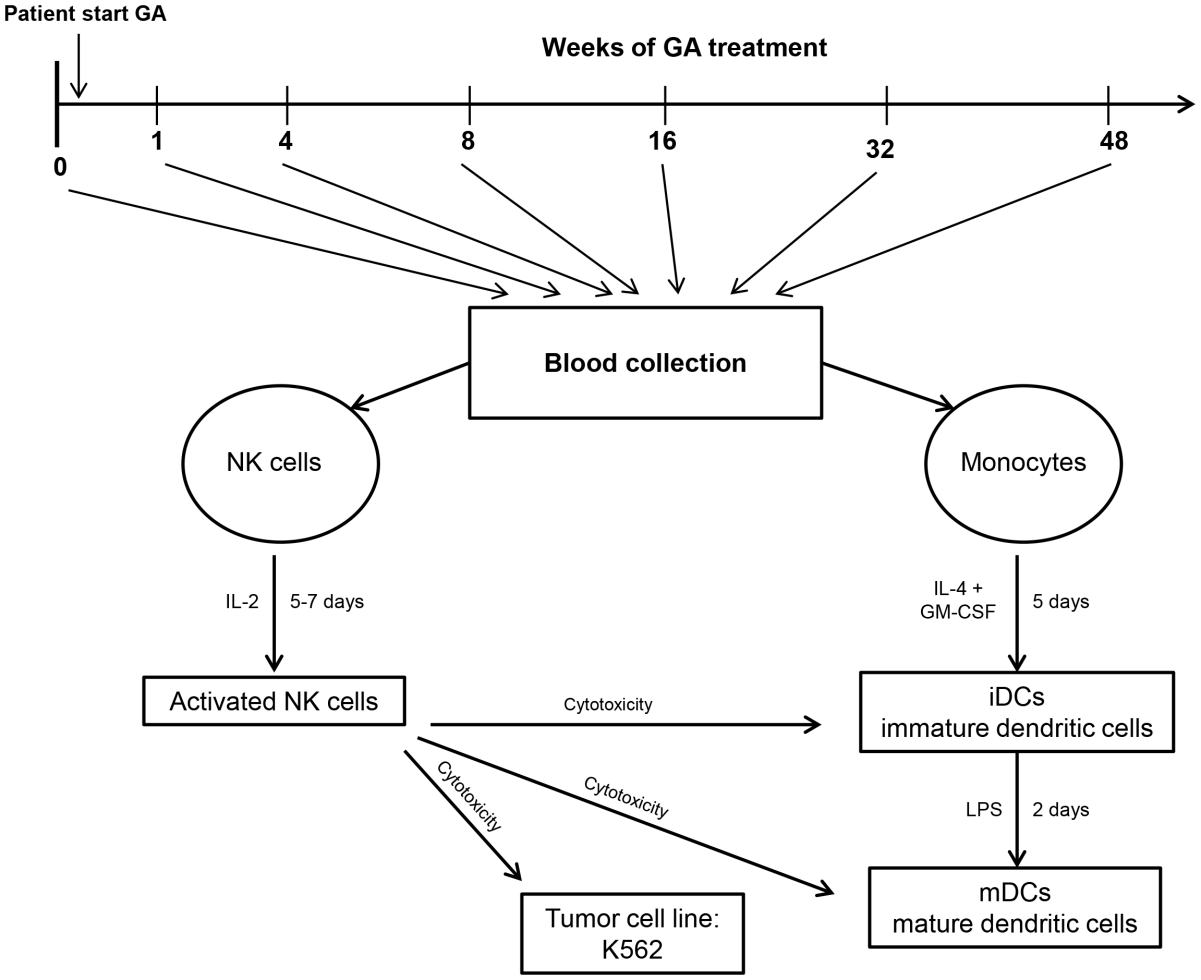
An overview of the study design. Patients were examined before initiation of treatment, and then followed for up to 48 weeks with repeated blood sampling (1, 4, 8, 16, 32 and 48 weeks after initiation of GA treatment). The procedures for isolation, maturation and activation of NK cells and DCs are described in the materials and methods section.

Human IL-2 activated NK cells were prepared using Histopaque-1077 (Sigma-Aldrich, Oslo, Norway) and RosetteSep human NK cell enrichment cocktail (Stemcell Technologies, SARL, Grenoble, France). NK cells were negatively selected by removing cells expressing CD3, CD4, CD19, CD36, CD66b, CD123 and glycophorin A. Purified NK cells were then placed in flasks at 1×10^6^/mL and 200 U/mL IL-2 (Peprotech, Rocky Hill NJ, USA), and then incubated at 37°C in a 5% CO_2_ incubator for 5 to 7 days. The efficacy of this method has been previously described [17]. Human monocytes were isolated using RosetteSep human monocyte enrichment cocktail (Stemcell Technologies), which negatively selects CD2, CD3, CD8, CD19, CD56, CD66b, CD123 and glycophorin A and leaves human monocytes intact. Cells were then cultured in petri dishes at 37°C in 5% CO_2_ incubator. Cell concentrations at 1×10^6^/mL were incubated with 6 ng/mL IL-4 and 25 ng/mL GM-CSF (Peprotech) for 5 days to generate iDCs. Mature dendritic cells (mDCs) were generated by adding 1 µg/mL LPS (Sigma-Aldrich), to iDCs for additional 24 hours. Figure 1 shows the protocols used in this study.

### Flow Cytometry Analysis

Activated NK cells were washed, re-suspended in PBS containing 0.1% sodium azide and incubated for 45 min at 4°C with 1 µg/mL PE-conjugated mouse anti-human control IgG1, NKp30 (CD337), NKp44 (CD336), NKp46 (CD335), NKG2D (all from Beckton-Dickinson Pharmingen, San Diego CA, USA), or CD158 (R&D systems, Minneapolis MN, USA), and FITC-conjugated mouse anti-human IgG2B (R&D systems) as a control. The marked cells were then washed twice and analyzed in a flow cytometer (FACSCalibur, Beckton Dickinson Biosciences, San Jose, CA). Immature and mature DCs were washed, re-suspended in PBS containing 0.1% sodium azide and incubated for 45 min at 4°C with 1 µg/mL FITC-conjugated anti-human IgG1, anti-HLA-DR, anti-CD80, anti-CD83 or anti-CD86 (R&D systems). Additionally mDCs were incubated with FITC-conjugated anti-CCR6, anti-CCR7, anti-IgG2A or anti-IgG2B (R&D systems). For labeling with unconjugated mouse anti-human IgG, anti-HLA-E and anti-HLA-I (eBiosciences, San Diego CA, USA), these cells were first incubated with unconjugated antibodies for 45 min at 4°C, washed twice and then labeled with FITC-conjugated goat anti-mouse (BD Pharmingen) and incubated for another 45 min at 4°C. Labeled cells were then washed twice and analyzed in the flow cytometer. Sample gating was individually set for each patient when treatment started, and used throughout the follow-up periods.

### Cytotoxicity Assay

Autologous iDCs and mDCs, as well as the leukemia cell line K562 cells (CCL-243 obtained from American Type Culture Collection “ATCC”, Manassas, Virginia, USA) were used as targets. These cells were incubated at 1×10^6^ cells/mL with 5 µg/mL calcein-AM (Teflabs, Austin TX, USA). NK cells were incubated with target cells at 5∶1 effector:target ratio in 12 wells in 96-well flat bottom plates (Corning Inc., NY, USA). The plates were centrifuged at 500 rpm for 5 min and incubated for 4 h at 37°C in 5% CO_2_. After incubation, the plates were centrifuged, and cell medium was replaced with PBS (Sigma-Aldrich). Fluorescence intensity of remaining calcein-AM loaded target cells was measured with BioTek FLX plate reader (Bio-Tek Instruments Inc., Winooski VT, USA), using 485/528 nm fluorescence filters. Percent cytotoxicity was calculated as previously described [18].

### Statistical Analysis

P values for the cytotoxicity assay replicates were calculated using the student’s t-test. Data from samples collected before and after start of treatment were compared for each patient. For flow cytometry the geometric mean of fluorescence intensity was calculated using the program FlowJo (Tree Star Inc., Ashland OR, USA).

## Results

### Effects of GA Treatment on the Ability of NK Cells to Kill Tumor Target Cells

**Figure 2:**
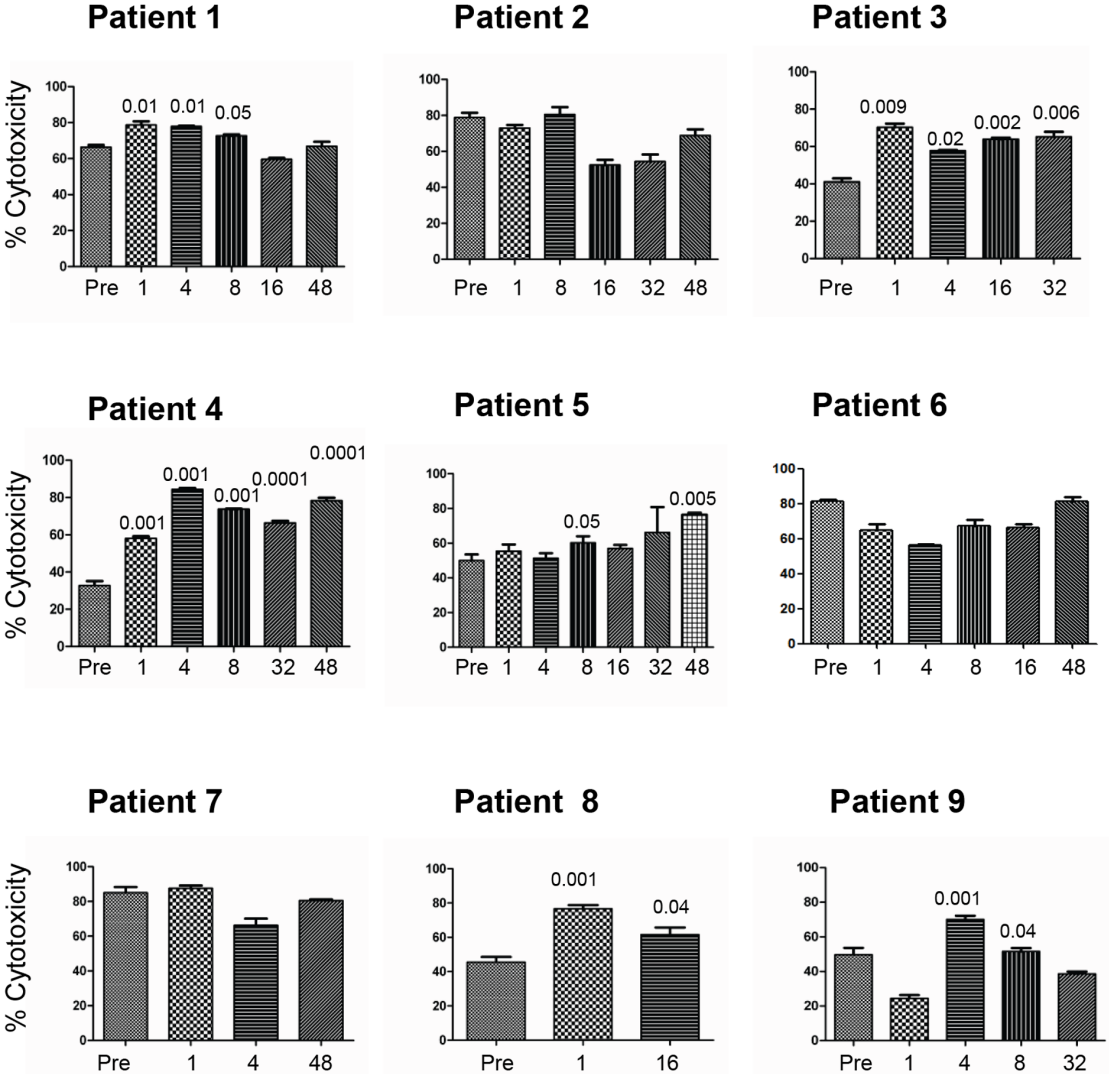
GA treatment of RRMS patients increases NK cell lysis of tumor target cells. NK cells were isolated from MS patients either pre-treatment (Pre) or after several weeks of treatment (ranging from 1–48 weeks). These cells were activated in vitro for five days and then examined for their ability to lyse the leukemic cell line K562. Percent cytotoxicity is shown as mean ± SEM of four parallel experiments done from cells isolated from the same patient. P values compared lysis of tumor cells by NK cells isolated from GA-treated patients before (Pre) and weeks after treatment.

We first aimed to examine the effects of GA treatment on the ability of NK cells to lyse the NK sensitive K562 tumor target cells. For this purpose, blood samples were collected from MS patients before start of treatment and at six time points for up to 48 weeks after initiation of GA treatment, and the ability of NK cells to lyse K562 before and after treatment was compared. In patient 1, there was enhancement of NK cell lysis of K562 at week 1, 4 and 8 (Figure 2). However, we observed no enhancement in patients 2, 6 and 7. In contrast, NK cells lysis of K562 in patients 3 and 4 increased significantly at all time points during therapy (Figure 2). There was a significant increase in NK cell lysis of K562 in patient 5 after 8 and 48 weeks of therapy. Samples from patient 8 were examined in blood collected weeks 1 and 16 after start of therapy, and NK cell lysis of K562 was significantly enhanced. NK cells collected after 4 and 8 weeks from patient 9 also significantly lysed K562.

### Effects of GA Treatment on the Ability of NK Cells to Kill Immature Dendritic Cells (iDCs)

**Figure 3:**
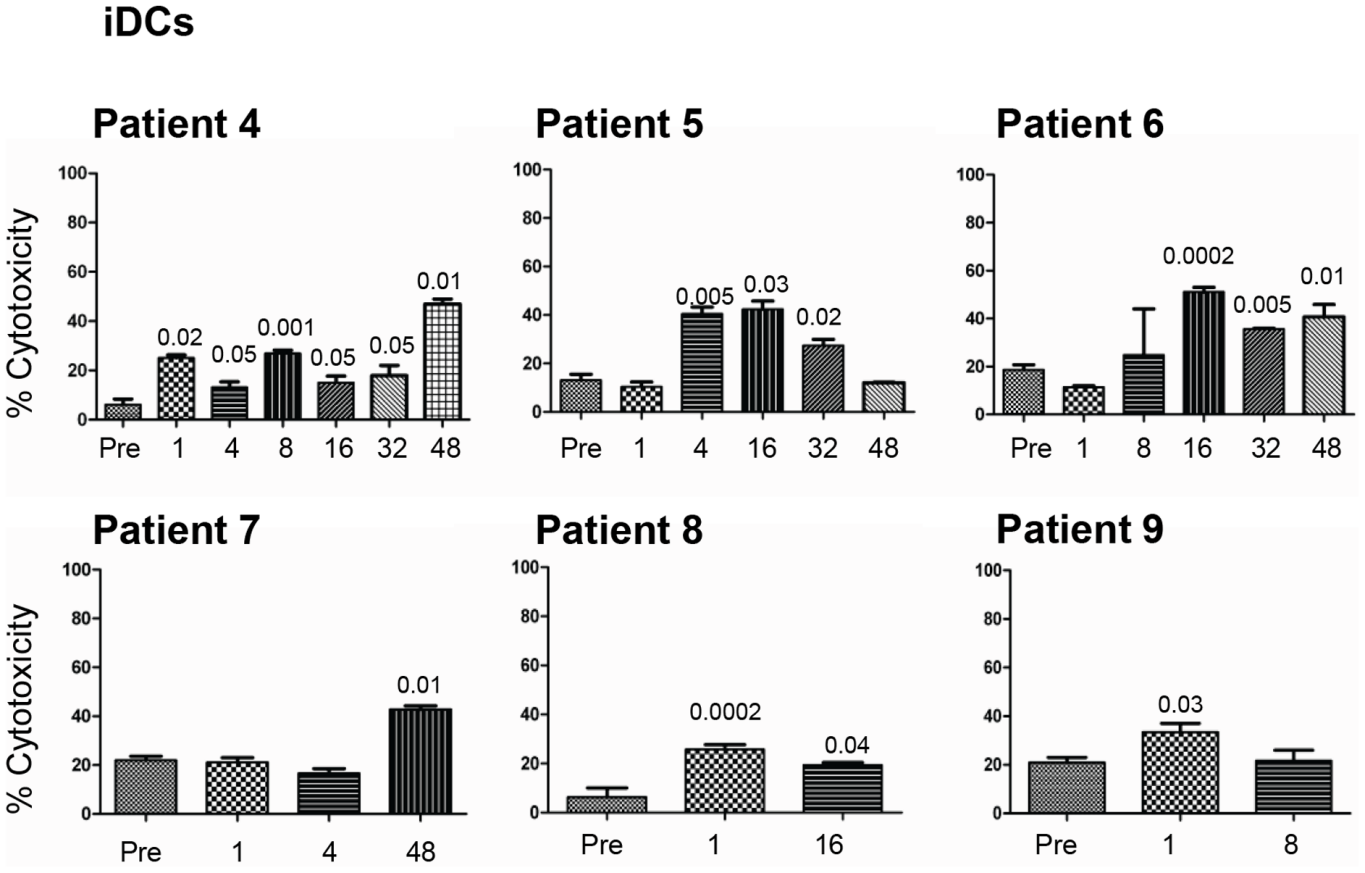
GA treatment of RRMS patients increases NK cell lysis of autologous monocyte-derived iDC and mDC target cells. NK cells were isolated from MS patients either pre-treatment (Pre) or weeks after treatment (ranging from 1–48 weeks). These cells were activated in vitro for five days and then examined for their ability to lyse iDCs. Percent cytotoxicity is shown as mean ± SEM of four parallel experiments done on cells isolated from the same patient. P values compared lysis of tumor cells by NK cells isolated from GA-treated patients before (Pre) and weeks after treatment.

Next we compared the ability of NK cells to lyse autologous iDCs before and during GA treatment. Significant increase in NK cell lysis of autologous iDCs of patient 4 was observed at all time points examined (Figure 3). We also found a significant enhancement of NK cell lysis of autologous iDCs isolated from patient 5 after 4, 16 and 32 weeks of therapy. Similarly, a significant increase in NK cell lysis of iDCs was observed after 16, 32 and 48 weeks in patient 6. Such an enhancement was only observed after 48 weeks in patient 7. For patient 8, the increase was noted after 1 and 16 weeks, and in patient 9 an increase was significant after 1 week of treatment (Figure 3).

**Figure 4:**
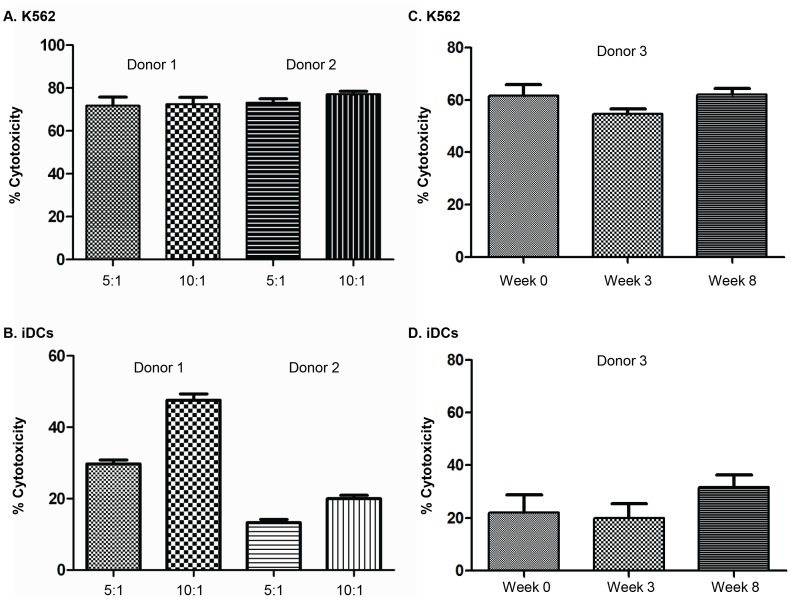
Assessments of experimental variability of the cytotoxicity assays. NK cells were isolated from three healthy individuals. These cells were activated in vitro for five days and then examined for their ability to lyse iDCs and K562 tumor cells. Percent cytotoxicity is shown as mean ± SEM. A) and B) show assays done from two healthy donors with two different E:T ratios. C) and D) show the results from a third healthy donor with repeated samples at baseline, or 3 and 8 weeks later.

It was difficult to isolate enough NK cells from MS patients to conduct several effector:target cell ratios for lysis of both K562 and iDCs. However, we performed these experiments using NK cells from two healthy donors, and observed that NK cells killed K562 with similar efficacy at both 10∶1 and 5∶1 E:T ratios (Fig. 4A). Lysis of iDCs was more potent at the 10∶1 as compared to the 5∶1 E:T cell ratio (Fig. 4B). In order to rule out the possibility that variation in experimental settings or other temporal variation might have influenced the results, we also investigated the effects of NK cell lysis of K562 and iDCs isolated from a healthy donor at time zero, 3 and 8 weeks later. These results were analyzed simultaneously with NK cells isolated from GA-treated patients. We found indications that there were no significant differences in NK cell lysis of either K562 (Figure 4C) or iDCs (Figure 4D) at these time points, which makes it unlikely that variations in experimental settings might have influenced the results.

### Effects of GA Treatment on the Ability of NK Cells to Kill Mature Dendritic Cells (mDCs)

**Figure 5:**
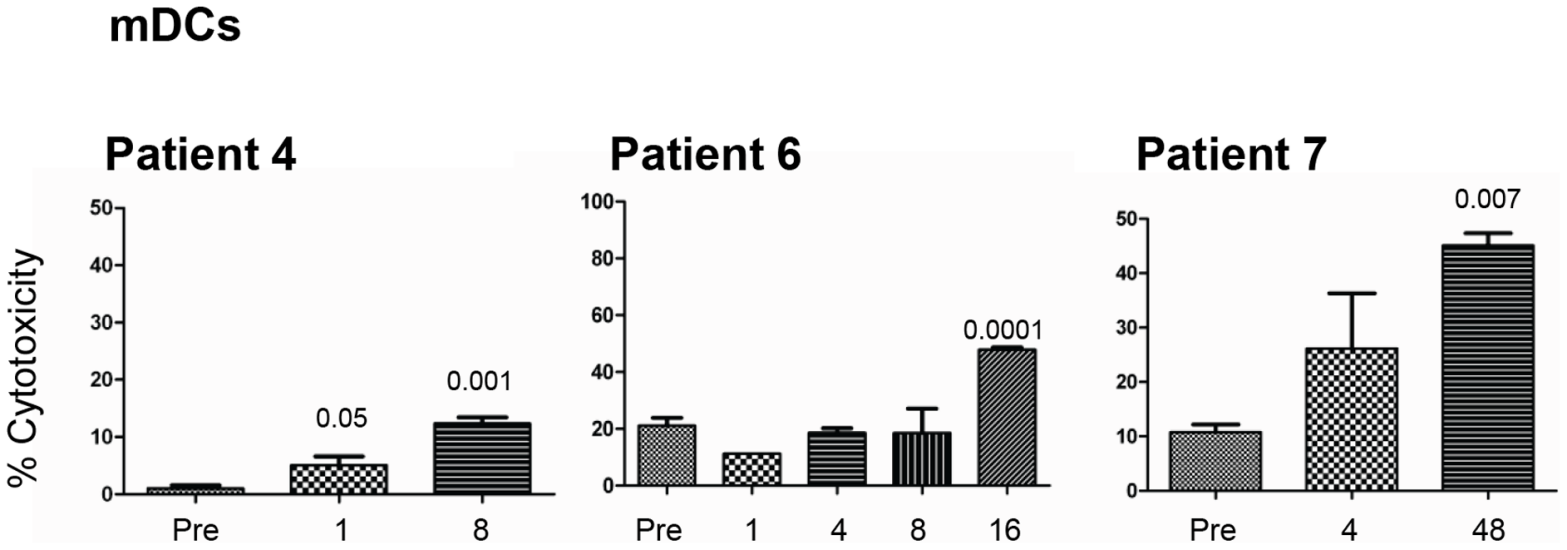
GA treatment of RRMS patients increases NK cell lysis of autologous monocyte-derived mDC target cells. NK cells were isolated from MS patients either pre-treatment (Pre) or weeks after treatment (ranging from 1–48 weeks). These cells were activated in vitro for seven days and then examined for their ability to lyse mDCs. Percent cytotoxicity is shown as mean ± SEM of four parallel experiments done on cells isolated from the same patient. P values compared lysis of tumor cells by NK cells isolated from GA-treated patients before (Pre) and weeks after treatment.

In this category only three patients were examined. A significant enhancement of NK cell lysis against mDCs was observed after 1 and 8 weeks of treatment in patient 4; after 16 weeks in patient 6; and after 48 weeks in patient 7 (Figure 5).

### Effects of GA Treatment on the Expression of NK Cell Surface Molecules

**Figure 6:**
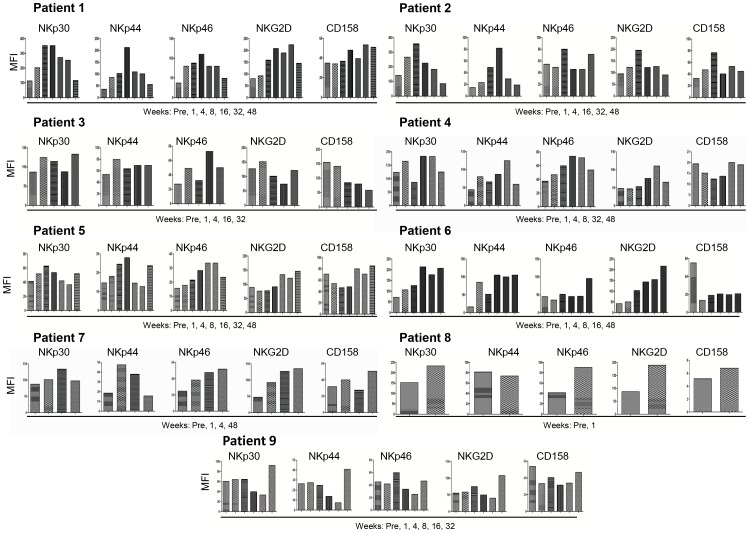
IL-2 activated NK cells from MS patients treated with GA show increased expression of activating cytotoxicity receptors. NK cells from MS patients were activated with IL-2 in vitro for five days and then examined for the expression of activating receptors NKp30, NKp44, NKp46 and NKG2D, as well as the inhibitory receptor CD158. Figures show comparisons of receptor expression before (Pre) and weeks after treatment start with GA. Mean fluorescence intensity (MFI) of 10,000 analyzed cells is shown.

To understand in detail the ability of NK cells to lyse target cells, we examined the effects of GA treatment on the expression of NK cytotoxicity receptors NKp30, NKp44, NKp46, and NKG2D as well as the inhibitory receptor CD158. Compared to baseline, there was enhanced expression of NKp30, NKp44, NKp46 and NKG2D at all time points after blood collection in patient 1 (Figure 6). NKp30 expression was increased after 1, 4, 16 and 32 weeks, whereas the expression of NKp44 was up-regulated at all time points in patient 2. NKp46 expression of this patient was only enhanced after 4 and 48 weeks of therapy, whereas a tendency of increase in NKG2D expression was noted after 4 weeks. Some up-regulation was noted in the expression of NKp30 and NKp44 after 1, 4 and 32 weeks of therapy in patient 3; whereas NKp46 expression was increased after 1 and 16 weeks. Of note, the inhibitory receptor CD158 expression was reduced at all time points, and in particular after 4, 16 and 32 weeks in patient 3 (Figure 6). NKp30 expression was enhanced after 1, 8, and 32 weeks in patient 4, whereas NKp44 and NKp46 expression was up-regulated at all time points during therapy, and NKG2D expression was enhanced after 32 weeks. Similar to patient 3, the expression of CD158 was reduced after 1, 4 and 8 weeks in patient 4 (Figure 6). An increase in the expression of NKp30 and NKp44 was noted after 1, 4, 8 and 48 weeks in patient 5, whereas NKp46 expression was up-regulated after 8, 16 and 32 weeks, and NKG2D expression was up-regulated after 16, 32 and 48 weeks. NKp30, NKp44 and NKG2D (except after one week) expression was higher than before treating patient 6. However, NKp46 was increased only after 48 weeks and in contrast CD158 expression was reduced at all weeks post treatment (Figure 6). NKp30 or NKp44 expression was up-regulated after 4 weeks in patient 7, whereas NKp46 and NKG2D expression was higher 1, 4 and 48 weeks later. The expression of NK cell receptors in patient 8 was examined only before treatment and after 1 week; except for NKp44 all other molecules expression was higher after 1 week. NKp30, NKp44 and NKG2D expression was higher after 32 weeks than before GA treatment in patient 9, whereas CD158 was lower at almost all weeks post treatment (Figure 6). To correlate the results obtained with the MFI on the expression of killer inhibitory receptors (KIR), we investigated the percentage of CD158^+^ NK cells. The results show that percentages of CD158^+^ NK cells are well correlated with the MFIs of this receptor (Figure S1).

### Effects of GA Treatment on the Expression of iDCs Surface Molecules

**Figure 7:**
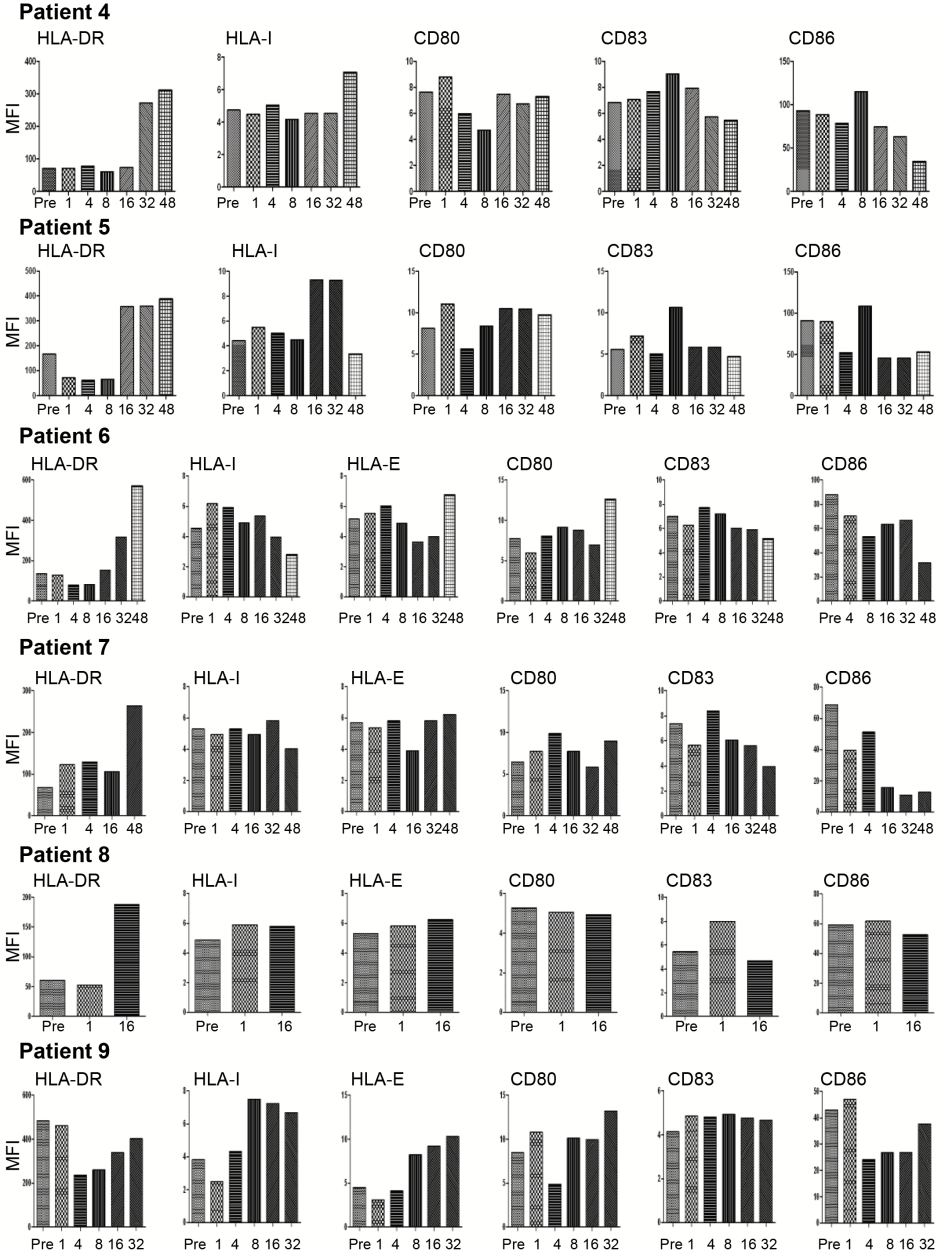
Treatment with GA changes expression of surface molecules in iDCs from RRMS patients. The expression of HLA-DR, HLA-I, HLA-E as well as the co-stimulatory molecules CD80, CD83 or CD86 was analyzed by flow cytometry of iDCs from RRMS patients before (pre) and weeks after treatment-start. Mean fluorescence intensity (MFI) of 10000 analyzed cells is shown.

Compared to baseline, HLA-DR and HLA-I expression on the surface of iDCs was increased after 32 and 48 weeks of therapy in patient 4. There was some decrease in the expression of the co-stimulatory molecules CD83 and CD86 after prolonged therapy, e.g. after 32 and 48 weeks (Figure 5). HLA-DR and HLA-I expression was also increased after 16 and 32 weeks (also after week 48 for HLA-DR) in patient 5. CD83 and CD86 expression was reduced on the surface of iDCs in patient 5 after 4, 16, 32 and 48 weeks (Figure 7). Similarly, HLA-DR expression on the surface of iDCs was up-regulated 32 and 48 weeks in patient 6, whereas HLA-I expression was enhanced after 1, 4, 8 and 16 weeks (Figure 7). Of the co-stimulatory molecules, the expression of CD83 and CD86 was reduced on iDCs after 48 weeks in patient 6. The trend for increased HLA-DR expression at a later time point (week 48) was also noted in patient 7. The expression of co-stimulatory molecules on iDCs, in particular CD86, was reduced at most weeks post-therapy (Figure 7). The expression of HLA-DR, but not any other molecule, was up-regulated after 16 weeks of treatment in patient 8 (Figure 7). The only exception to the findings of increased HLA-DR expression was noted in patient 9. The iDCs of this patient showed also reduced expression of CD86 after 4, 8, 16 and 32 weeks of treatment (Figure 7).

### Effects of GA Treatment on the Expression of Surface Molecules on mDCs

**Figure 8:**
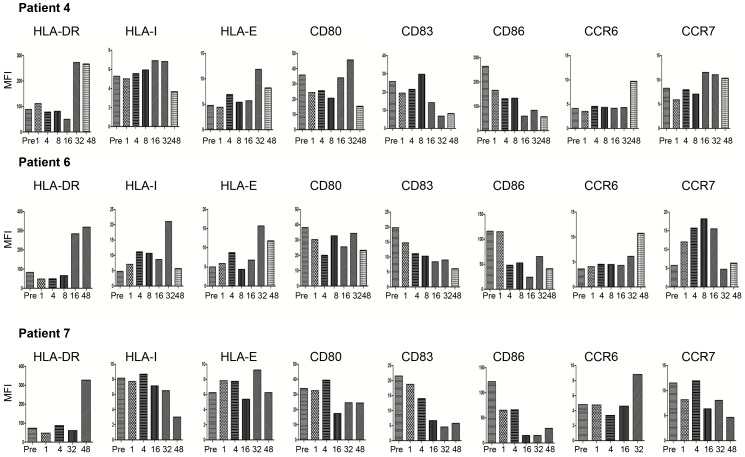
Treatment with GA changes expression of surface molecules in mDCs from RRMS patients. The expression of HLA-DR, HLA-I, HLA-E as well as co-stimulatory molecules CD80, CD83 or CD86 was analyzed with flow cytometry of mDCs from RRMS patient before (Pre) and weeks after treatment start. Mean fluorescence intensity (MFI) of 10000 analyzed cells is shown.

Finally, we examined the expression of surface molecules on mDCs. Compared to baseline, HLA-DR expression increased during treatment, which was recorded between weeks 16 and 48 (patients 4, 6 and 7). The expression of HLA-I was also enhanced, particularly after week 32 in patients 4 and 6, but not in patient 7 (Figure 8). Regarding the co-stimulatory molecules, there was an increase in the expression of CD80 after week 32 in patient 4. The expression of CD83 and CD86 was reduced particularly at later time points, i.e. between weeks 16 and 48. The chemokine receptors that are important for the circulation of DCs (CCR6) and for their extravasations into the lymph nodes (CCR7) were also examined. In all three patients the expression of CCR6 was up-regulated after 32 and 48 weeks of GA treatment. However, no consistent findings were observed for CCR7 expression (Figure 8).

## Discussion

By studying blood samples from RRMS patients collected before and during the first year of GA treatment, we found that GA increased NK cell cytotoxic activity towards the tumor cell line K562 as well as autologous iDCs and mDCs. The expression of activating NK cytotoxicity receptors was increased, whereas the expression of inhibitory molecules was reduced on the surface of NK cells. This correlated closely with the ability of NK cells to lyse DCs rather than K562 cells. For example, NK cells isolated from patients 6 and 7 killed both iDCs and mDCs, but failed to lyse K562 cells. This was in line with the up-regulation of NK cell cytotoxicity receptors on the surface of NK cells whereas the inhibitory molecule CD158 was down-regulated in these two patients.

It has previously been suggested that NK cells play a primary role in ameliorating EAE, both due to their accumulation within target organs and because depletion of these cells aggravated the disease [19]. Depletion of NK cells before immunization has been reported to result in more severe EAE, increased T cell proliferation and production of Th1 cytokines, indicating that NK cells inhibit T cell proliferation triggered by CNS autoantigens [20]. Matsumoto et al. observed that NK cells were recruited from the spleen into the CNS of EAE rats. They also observed that treatment of animals with anti-3.2.3 antibody or anti-asialo GM1 antibodies that deplete NK cells exacerbated EAE [21]. Further, administration of an antibody that relieves NK cells from inhibition resulted in increased NK cell lytic activity in the CNS with consequent complete remission from EAE [22]. Although the role of NK cells is not completely clear, it may be that these cells represent a potential drug target in autoimmune diseases such as MS.

We have previously reported that GA enhances in vitro human NK cell lysis of autologous and allogeneic iDCs and mDC [15]. These findings were in line with other observations showing that GA ameliorated clinical EAE scores, and that NK cells from these mice lysed both iDCs and mDCs [16]. These results indicated that one possible mechanism of GA inhibition may be to activate NK cells to lyse DCs (reviewed in [23]). Similarly, recent observations have shown that mitoxantrone, another drug used for treating MS, induces NK cell enrichment and maturation, which was associated with improved clinical response in secondary progressive MS [24].

Here, we examined the effects of NK cells lysis of tumor target cells K562 as well as iDCs and mDCs in nine MS patients treated with GA for up to 48 weeks after start of treatment. NK cells isolated from six of these patients showed significantly enhanced lysis of K562 tumor target cells, which occurred at somewhat different time-points. However, NK cells isolated from all patients killed iDCs and mDCs. These findings confirm our previous in vitro results [15] as well as results obtained from mice with EAE [16]. We also noted that when cytotoxicity increased, the expression of the inhibitory receptor CD158 decreased. However, we did not observe synchronicity between increased expression of NK cell activating receptors and down-regulation of CD158 among all patients receiving GA, but the modulatory expression of this molecule rather occurred at different times in therapy of each patient. Others have shown that activating receptor NKp44 is up-regulated upon activation of NK cells with IL-2 or IL-15 [25], whereas the other cytolytic receptors NKp30 and NKp46 are ubiquitously expressed on all NK cells [26], [27]. NKG2D is a C-type lectin stimulatory receptor which recognizes cells that contain molecules expressed on cells after stress. In humans, these molecules are known as UL-16 binding protein (ULBP1, 2 and 3), or MHC class I chain-related (MICA/MICB) antigens [28].

We also noticed variations in the expression of MHC and co-stimulatory molecules on the surfaces of DCs after GA treatment. The significance of increased or decreased surface expression of HLA and co-stimulatory molecules on iDCs and mDCs is not clear at the present time, but it has been reported that treatment of mDCs with GA in vitro reduces the expression of HLA-I and HLA-DR on the surface of these cells, correlated with the ability of NK cells to lyse them [15]. It has also been reported that anti-CD83 antibody inhibits NK cell lysis of iDCs after exposure to GA, whereas anti-CD86, anti-CD83, anti-HLA-DR and anti-HLA-I antibodies inhibit in vitro GA-enhanced NK cell lysis of mDCs [15]. Whether MHC and co-stimulatory molecules have similar roles on the activity of GA in MS patients is not yet known. Although we have no formal proof, it is plausible that down-regulation of the co-stimulatory molecules CD83 and CD86 on the surface of iDCs and mDCs upon GA treatment may reduce their ability to present antigens to autoreactive T cells.

Interestingly, we observed that CCR6 expression was increased on the surface of mDCs, particularly after 36 or 48 weeks of therapy. CCR6 induces the migration of Th17 cells into the gut [29], and facilitates the recruitment of Th17 cells into the CNS via the CSF [30]. It may be that CCR6 also guides mDCs into the CSF. Dendritic cells are presumed to have important roles in MS, since myeloid DCs in EAE accumulate in the CNS, where they present myelin autoantigens to CD4^+^ T cells that may differentiate into Th17 cells [5]. Hence, targeting the accumulation of DCs in the inflamed brain could be an important therapeutic effect of GA.

In summary, we provide comprehensive data regarding the activity, phenotypic expression and interaction of two of the most important cell types of innate immunity, i.e. NK cells and DCs, in MS patients treated with GA. The fact that the included patients were followed up to one year post-therapy, and the various effects of the cells examined are important aspects in this study. We show that GA treatment promotes NK cells to kill both iDCs and mDCs, correlating with increased expression of NK activating molecules and down-regulation of inhibitory molecules on the surfaces of NK cells. These results suggest that GA treatment results in the destruction of antigen presenting cells, and consequently impedes presentation of antigens to autoreactive T cells. Although the number of patients examined was small in our study, the results may lead to novel hypotheses and could pave the way for better understanding of the effect of GA in MS treatment.

## Supporting Information

Figure S1 **Percentage of IL-2 activated NK cells expressing CD158.** NK cells from patients were activated with IL-2 in vitro for five days and then examined for the expression of the inhibitory receptor CD158. The figure shows comparisons of receptor expression before (Pre) and weeks after treatment start with GA. Percentages of cells expressing the surface molecule is shown.
(TIF)
