# Supplementary figures and images for: A One Year Follow-Up Study of Natural Killer and Dendritic Cells Activities in Multiple Sclerosis Patients Receiving Glatiramer Acetate (GA)
Source: PLoS One. 2013 Apr 22;8(4):e62237. doi: 10.1371/journal.pone.0062237 (PMC3632560; doi:10.1371/journal.pone.0062237)

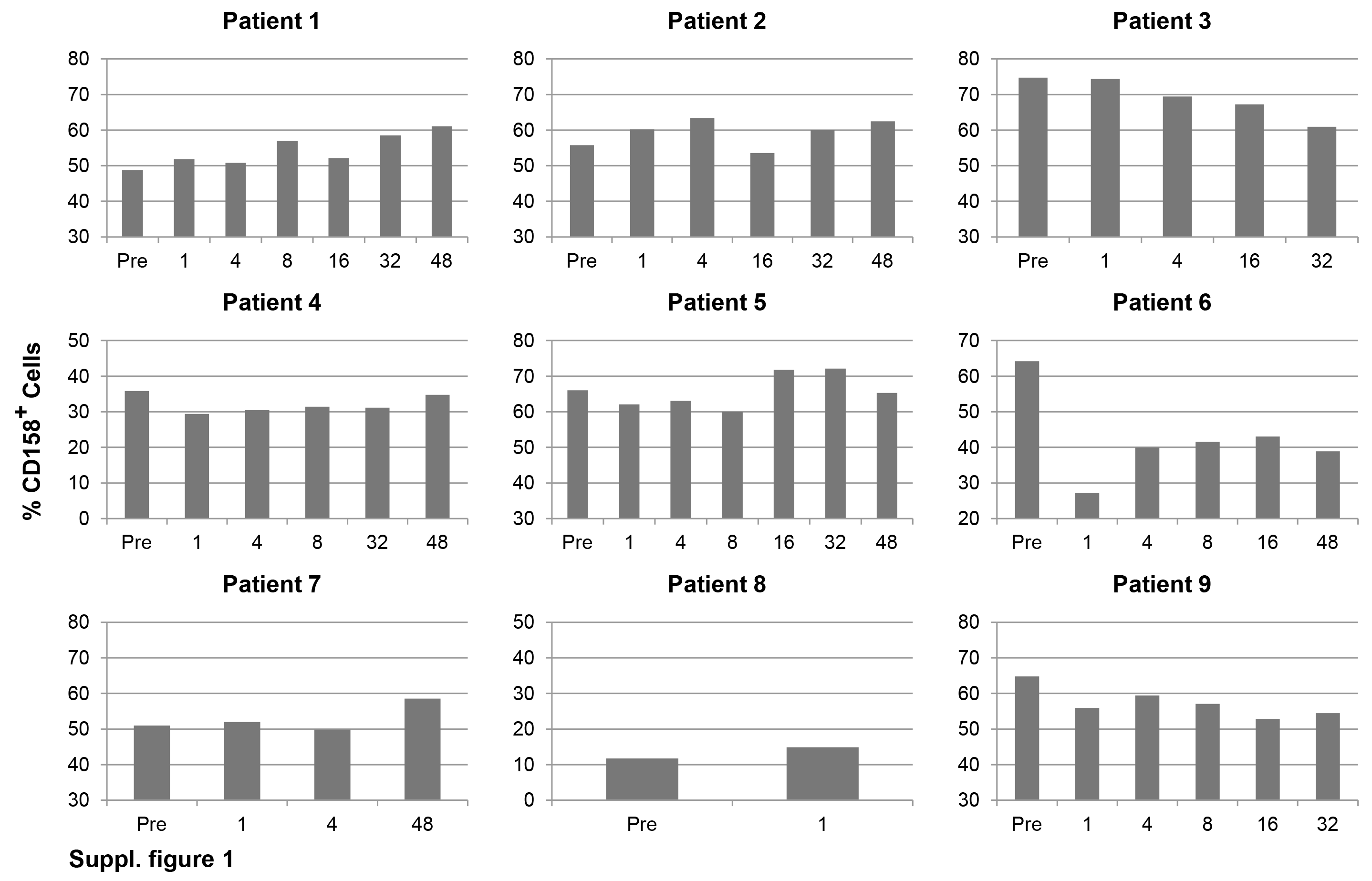

Supplement: Figure S1 — Percentage of IL-2 activated NK cells expressing CD158. NK cells from patients were activated with IL-2 in vitro for five days and then examined for the expression of the inhibitory receptor CD158. The figure shows comparisons of receptor expression before (Pre) and weeks after treatment start with GA. Percentages of cells expressing the surface molecule is shown. (TIF) [file pone.0062237.s001.tif]
